# Iris Vascular Malformation with 360-Degree Iridocorneal Angle Affectation

**DOI:** 10.1155/2020/5913636

**Published:** 2020-06-02

**Authors:** Cristina Calleja-García, Jorge Suárez-Baraza, Enrique Mencía-Gutiérrez

**Affiliations:** Ophthalmology Department, 12 de Octubre Hospital, Complutense University, 28041 Madrid, Spain

## Abstract

Vascular iris lesions are rare and can sometimes be associated to systemic vascular lesions. They usually cause spontaneous recurrent hyphema. The differential diagnosis should be considered primarily with iris rubeosis and with highly vascular tumors as iris melanoma. Generally, vascular lesions of hemangioma type are located in the iris without extension to iridocorneal angle. We present a case of a 77-year-old male with an iris vascular lesion suggestive of racemose hemangioma, who is asymptomatic, and with 360-degree iridocorneal angle affectation showing no lesions at any other location.

## 1. Introduction

There are a variety of tumors and iridian lesions that range from cystic tumors to even solid melanocytic and nonmelanocytic tumors. According to Shields et al., the nonmelanocytic lesions constitute 11% of iridian tumors, and of these, the vascular type constitutes 2% [1]. Different types of iridian hemangiomas can be distinguished: racemose, cavernous, capillary, and pupil rim microhemangioma, and they must be distinguished from other arteriovenous alterations such as iris rubeosis among others [2].

## 2. Case Report

**Figure 1:**
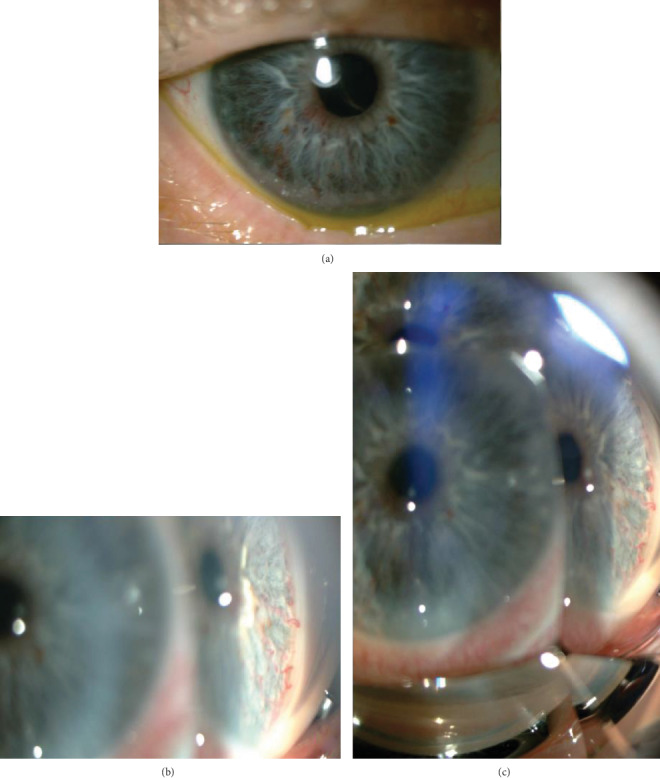
(a) Radial iridian vascular engorgement at 5 to 7 o'clock location in RE. (b, c) Gonioscopic examination shows a graded IV open angle with a vascular racemose net of 360 degrees in the iridocorneal angle.

**Figure 2:**
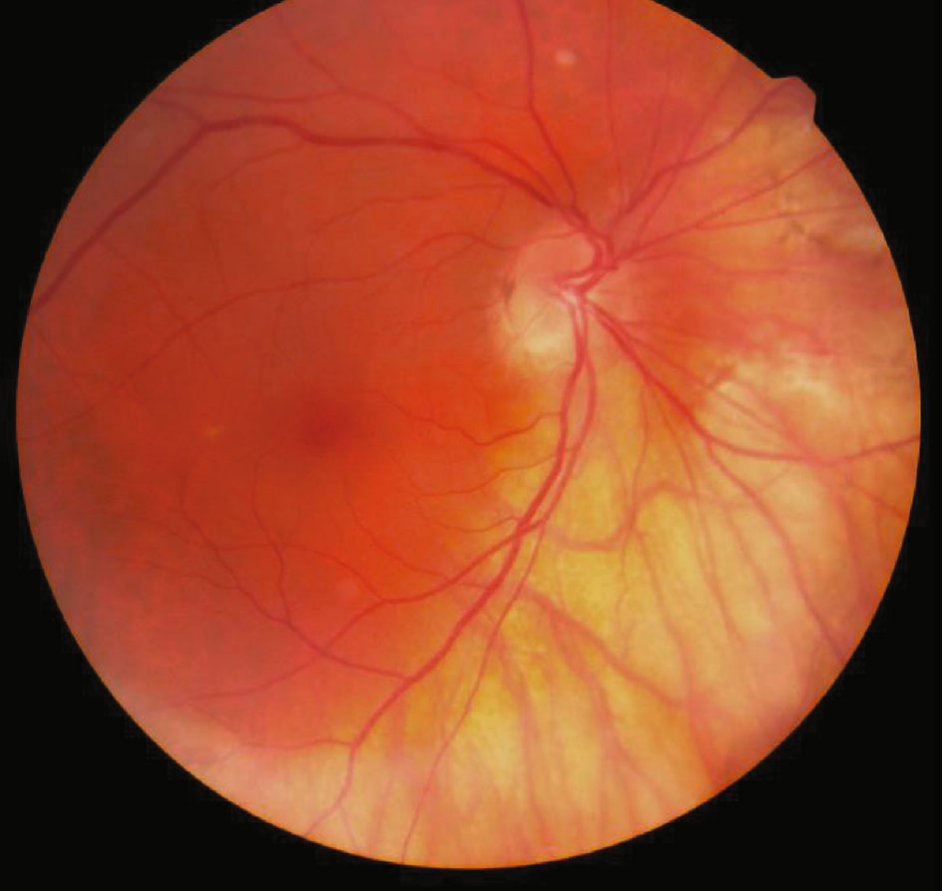
RE ocular fundus shows an oblique papilla and myopic coriorretinitis without other findings.

A 77-year-old male was referred for a possible study of unilateral iris rubeosis in right eye (RE) found in a routine exploration previous to cataract surgery. The patient was asymptomatic and did not refer any previous ocular pathology except divergent strabismus since childhood with left eye amblyopia. The patient had a personal history of arterial hypertension with renal repercussion, metabolic syndrome, auricular fibrillation, prostatic syndrome, and moderate chronic obstructive pulmonary emphysema. In the exploration, he presented a visual acuity (VA) (decimal scale) of the RE of 0.5 and in the left eye (LE) of 0.15, as well as divergent strabismus. In a slit lamp, we observed radial iridian vascular engorgement at 5 to 7 o'clock location in RE. The vessels seem to originate from the iris stroma and then extended circumferentially (Figure 1(a)). Gonioscopic examination showed a graded IV open angle with a vascular racemose net of 360 degrees in the iridocorneal angle (Figures 1(b) and 1(c)). No sentinel vessels were found at the episcleral level, and the presence of masses at other sites of the iris was ruled out. Intraocular pressure in the RE was 10 mmHg and in the LE, 12 mmHg. LE examination was normal. Ocular fundus showed in the RE an oblique papilla, myopic coriorretinitis, without exudates, hemorrhages, or neovascularization (Figure 2). There were not any clinical signs of pathology that could cause retinal ischemia such as diabetic retinopathy, central or branch vein, or central artery obstruction.

Peripheral tumor masses, such as choroidal melanoma, retinoblastoma, metastasis, or ciliary body lesions, were ruled out by fundus examination with indentation and maximum midriasis.

Supra-aortic trunk exam with ecoDoppler showed no other causes of ocular ischemia. No carotid stenosis with hemodynamic significance was observed. In the differential diagnosis, entities such as sickle-cell anemia, Takayasu disease, giant cell arteritis, or carotid-cavernous fistula were also proposed, but additional tests were not requested since they have low probability in the patient's clinical context.

**Figure 3:**
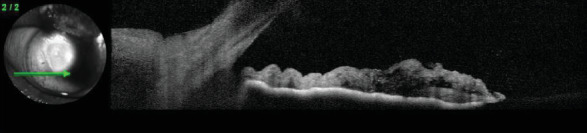
RE anterior segment optic coherence tomography shows intrastromal hyporeflective spaces corresponding to the iris vessels.

An anterior segment optic coherence tomography was performed to refine the characterization of the lesion, and poorly defined intrastromal hyporeflective spaces were observed coinciding with the lumen of the vessels (Figure 3).

**Figure 4:**
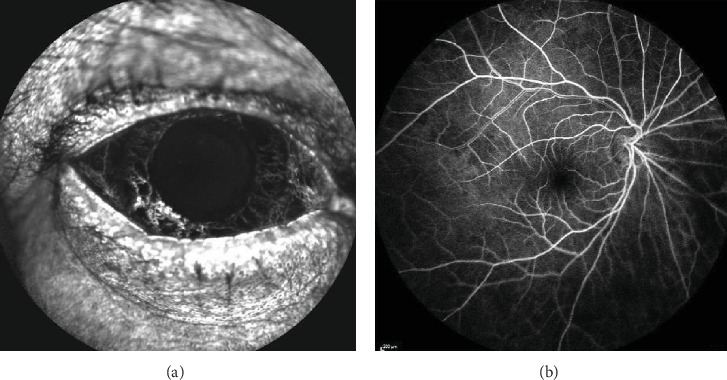
(a, b) RE fluorescein angiography shows an early filling of the vessels without contrast diffusion in the iris, angle, and retina vessels.

For the differential diagnosis between neovascularization and tumor/vascular malformation, a fluorescein angiography (FA) was performed and normal contrast uptake was demonstrated without extravasation of the latter at the level of the iridian, iridocorneal angle, and retinal vessels (Figures 4(a) and 4 (b)). An early filling of the iridian vessels was observed without contrast diffusion, thus ruling out retinal ischemia and neovessels.

## 3. Discussion

Vascular malformations of the iris are those in which there is a connection between an artery and a vein without an interposed capillary plexus, and they are very rare. They can cause spontaneous hyphemas associated with ocular hypertension. These lesions must be distinguished from other arteriovenous alterations such as iris rubeosis, juvenile xanthogranuloma, iris varix, and highly vascular melanoma [2]. Different types of iridian hemangiomas can be distinguished: racemose, cavernous, capillary, and pupil rim microhemangioma. The racemose hemangioma can be associated with the presence of dilated episcleral vessels that are related to the presence of a ciliary body melanoma [3]. As for the cavernous hemangioma, Shields et al. cast doubt on its existence since they consider that it can be part of a spectrum of lesions, including thrombosed iris varicose veins. However, these may be associated with lesions of the cavernous hemangioma at the brain level. Capillary hemangioma is extremely rare and is usually more frequent in childhood, associated with ipsilateral cutaneous lesions [3]. Iris microhemangiomas are the most frequent vascular lesions of the iris, and they are diagnosed when spontaneous hyphema is associated. This subtype of hemangioma is related by an unknown mechanism with myotonic dystrophy and with diabetes mellitus [3].

Shields et al. [2] reported a mean age of diagnosis of 55 years, all lesions being unilateral. They are more frequent in clear eyes, probably because they are easier to visualize, and are usually located in the temporal region, although there is no clear explanation for this. It is not clear if they are congenital or acquired lesions. Of the 14 cases of arteriovenous iris malformations studied by Shields et al., none of them were associated with systemic diseases/syndromes. In 50% of cases, a sentinel episcleral vessel was located, which could have been a sign of a melanoma of the underlying ciliary body, but in all cases, its presence was ruled out.

These vascular-type lesions must be distinguished from other arteriovenous alterations such as iris rubeosis. For this, it is essential to detect those risk factors for ocular ischemia: venous thrombosis, diabetic retinopathy, central retinal artery obstruction, ocular ischemia syndrome (carotid stenosis, Takayasu disease, giant cell arteritis, and carotid-cavernous fistula), vasculopathies such as sickle-cell anemia or Eales disease, and tumors (choroidal melanoma and highly vascular iris).

FA plays a fundamental role in detecting the presence of neovascularization and areas of retinal ischemia. In iridian vascular malformations, FA is characterized by an early hyperfluorescence that may also be visible later and, unlike rubeosis, it will not produce contrast extravasation. FA is a very useful but invasive test, so new diagnostic methods such as optical coherence tomography angiography allow us to perform a more precise exploration of the iridian vasculature in a noninvasive way [4].

In the literature, there have been no reported cases of iridian vascular lesions of the hemangioma type in which the vascular malformation extends 360 degrees through the iridocorneal angle. Shields et al. [3] did not report cases of hemangiomas with the extension of the iridocorneal angle. The study by de Corral et al. [5] describes a case of spontaneous hyphema due to an anomalous vessel in the iridocorneal angle that does not present hemangioma characteristics.

In general, the management of these lesions consists of observation without any specific treatment, since most of them are asymptomatic, as in this case. In cases where there has been spontaneous bleeding, improvement has been reported after treatment with argon laser photocoagulation applied directly over the vascular lesion [5]. In case of doubt or diagnoses of melanoma or when they cause recurrent hyphema, a sectoral iridectomy can be performed with excision of the lesion.

## 4. Conclusion

Vascular malformations of the iris are very rare and can sometimes be associated with vascular lesions at other locations; however, in our case, we describe the existence of a vascular malformation known as racemose hemangioma type iris, with 360-degree extension by the iridocorneal angle without any other lesions. These are asymptomatic lesions that require follow-up and a differential diagnosis, mainly with iris rubeosis and neovascular glaucoma. Tumors such as ciliary body melanoma, although rare, should be ruled out.

## Data Availability

The data sets used to support the findings of this study are available from the corresponding author upon reasonable request.
